# Engineered botulinum neurotoxin B with improved efficacy for targeting human receptors

**DOI:** 10.1038/s41467-017-00064-y

**Published:** 2017-07-03

**Authors:** Liang Tao, Lisheng Peng, Ronnie P.-A. Berntsson, Sai Man Liu, SunHyun Park, Feifan Yu, Christopher Boone, Shilpa Palan, Matthew Beard, Pierre-Etienne Chabrier, Pål Stenmark, Johannes Krupp, Min Dong

**Affiliations:** 1 Department of Urology, Boston Children’s Hospital, Department of Microbiology and Immunobiology, Department of Surgery, Harvard Medical School, 300 Longwood Avenue, Boston, Massachusetts 02115 USA; 2 Department of Neurology, The Third Affiliated Hospital of Sun Yat-sen University, No. 600 Tianhe Road, Guangzhou, Guangdong 510630 China; 3 Department of Biochemistry and Biophysics, Stockholm University, Stockholm, SE-106 91 Sweden; 4 Department of Medical Biochemistry and Biophysics, Umeå University, Umeå, SE-901 87 Sweden; 5 IPSEN Bioinnovation, Abingdon, OX14 4RY UK; 6 Division of Predictive Toxicological Research, Predictive model Research Center, Korea Institute of Toxicology, 141 Gajeong-ro, Yuseong-gu, Daejeon 34114 South Korea; 7 IPSEN Innovation, Les Ulis, 91940 France

## Abstract

Botulinum neurotoxin B is a Food and Drug Administration-approved therapeutic toxin. However, it has lower binding affinity toward the human version of its major receptor, synaptotagmin II (h-Syt II), compared to mouse Syt II, because of a residue difference. Increasing the binding affinity to h-Syt II may improve botulinum neurotoxin B’s therapeutic efficacy and reduce adverse effects. Here we utilized the bacterial adenylate cyclase two-hybrid method and carried out a saturation mutagenesis screen in the Syt II-binding pocket of botulinum neurotoxin B. The screen identifies E1191 as a key residue: replacing it with M/C/V/Q enhances botulinum neurotoxin B binding to human synaptotagmin II. Adding S1199Y/W or W1178Q as a secondary mutation further increases binding affinity. Mutant botulinum neurotoxin B containing E1191M/S1199Y exhibits ~11-fold higher efficacy in blocking neurotransmission than wild-type botulinum neurotoxin B in neurons expressing human synaptotagmin II, demonstrating that enhancing receptor binding increases the overall efficacy at functional levels. The engineered botulinum neurotoxin B provides a platform to develop therapeutic toxins with improved efficacy.

## Abstract (web summary)

Humans are less sensitive to the therapeutic effects of botulinum neurotoxin B (BoNT/B) than the animal models it is tested on due to differences between the human and the mouse receptors. Here, the authors engineer BoNT/B to improve its affinity to human receptors and enhance its therapeutic efficacy.

## Introduction

Botulinum neurotoxins (BoNTs) are a family of bacterial toxins with seven major serotypes (BoNT/A–G)^1–3^. They are composed of a light chain (LC, ~50 kDa) and a heavy chain (HC, ~100 kDa), connected via a disulfide bond. The HC includes two subdomains: a C-terminal receptor-binding domain (H_C_) and an N-terminal translocation domain (H_N_) that translocates the LC across endosomal membranes. Once released into the cytosol, the LC acts as a protease to cleave a set of neuronal proteins: BoNT/A, C, and E cleave a protein known as SNAP-25; BoNT/B, D, F, and G cleave the vesicle protein VAMP (also known as synaptobrevin); and BoNT/C also cleaves the plasma membrane protein syntaxin 1^1, 2, 4^. These three proteins are known as neuronal SNAREs (soluble NSF (N-ethylmaleimide-sensitive factor) attachment protein receptors), which form a complex that mediates fusion of synaptic vesicle membranes to plasma membranes^4–6^. Cleavage of any one of these three proteins blocks synaptic vesicle exocytosis and release of neurotransmitters.

A minute amount of BoNT can be utilized to attenuate neuronal activity in targeted regions, making BoNTs effective for treating a growing list of medical conditions, including muscle spasms, chronic pain, and overactive bladder problems^7–10^. Among the seven major serotypes of BoNTs, BoNT/A, and BoNT/B (more precisely, BoNT/A1 and BoNT/B1 among the many subtypes of BoNT/A and BoNT/B) have been approved by the Food and Drug Administration (FDA) for use in humans. Medical applications of BoNTs are remarkably effective and well tolerated, but some limitations and adverse effects have also been reported. The major limitation is the generation of neutralizing antibodies in patients in rare cases, which renders future treatment ineffective^11^. The major adverse effects are largely due to toxin diffusion into other regions of the body, causing a range of side effects^12^. These limitations and adverse effects have a significant negative impact on the quality of treatment.

BoNTs target neurons with high efficacy and specificity. The neuronal receptors for most BoNT serotypes have been identified in recent years. BoNT/B, G, and DC (a mosaic toxin) share two homologous synaptic vesicle proteins, synaptotagmin I and II (Syt I/II), as their receptors^13–20^. Another family of synaptic vesicle proteins, SV2, acts as receptors for BoNT/A, E, D, and potentially F^14, 21–26^. In addition to protein receptors, all BoNTs require gangliosides as co-receptors, which are abundant on neuronal surfaces^27^. Enhancing the ability of BoNTs to recognize their neuronal receptors will facilitate absorbance/uptake of toxins into neurons at the injection site, minimizing the possibility of immune response and toxin diffusion. It also reduces any potential off-target effects due to nonspecific entry into other cell types.

Recent studies reported that BoNT/B1 (simply referred to as BoNT/B thereafter) has lower binding affinity toward human Syt II (h-Syt II) compared to rat/mouse Syt II because of a single amino-acid change from phenylalanine (F) in rat/mouse Syt II to leucine (L) in h-Syt II at position 54^28, 29^. Humans are still susceptible to BoNT/B, as h-Syt I still contains an F residue at this position. However, Syt II appears to be the dominant isoform expressed in motor nerve terminals, and rodent Syt II also showed ~10-fold higher binding affinity toward BoNT/B than Syt I^30, 31^. Thus, human motor neurons might be less sensitive to BoNT/B compared to mouse motor neurons, which is consistent with a long-standing clinical observation that BoNT/B has to be used at ~60–100-fold higher doses than BoNT/A to achieve the same level of effects in patients^32, 33^.

Clinical studies using the present BoNT/B product suggest that it may have certain advantages over BoNT/A for treating disorders such as sialorrhea, hyperhidrosis, and some smooth muscle-related disorders^34, 35^. In addition, BoNT/B is needed as an alternative toxin for treating patients who have developed neutralizing antibodies against BoNT/A. A modified BoNT/B with the ability to bind h-Syt II would have superior therapeutic efficacy over current BoNT/B products for these applications. Furthermore, because neurons express more copies of synaptotagmin I/II than SV2^36, 37^, a modified BoNT/B may have greater binding and entry efficacy into human neurons compared to BoNT/A. By combining rational design and saturation mutagenesis, here we identified a series of point mutations in BoNT/B that increased its binding affinity to h-Syt II. We further demonstrated that full-length BoNT/B containing the designed mutations showed ~11-fold higher efficacy than wild-type (WT) BoNT/B in neurons that express only h-Syt II as the receptor. These studies demonstrate that enhancing receptor binding increases overall efficacy in blocking neurotransmission at functional levels, providing a general approach to improve the therapeutic efficacy of BoNTs.

## Results

### Screen for mutations that enhance BoNT/B binding to h-Syt II

**Fig. 1:**
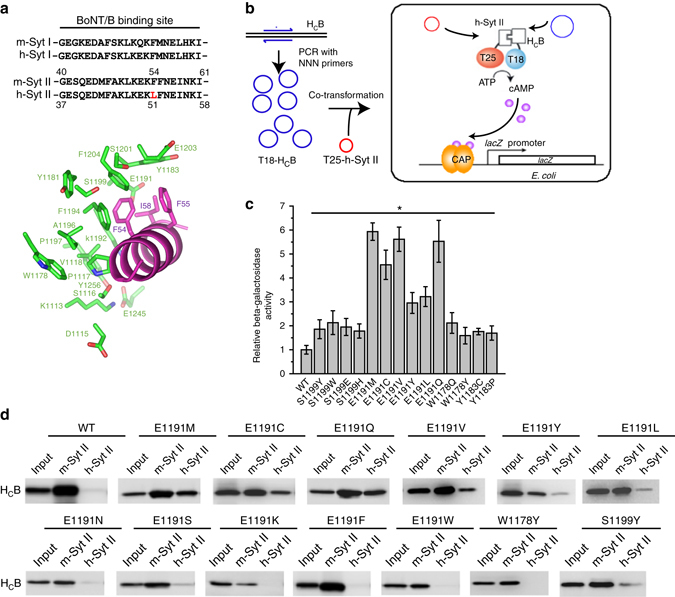
BACTH screen identifies mutations that enhance BoNT/B binding to h-Syt II. **a** Sequence alignment of the BoNT/B-binding site in mouse Syt I (m-Syt I), h-Syt I, h-Syt II, and mouse Syt II (m-Syt II), and a close-up view of the interface between H_C_B and Syt II. Targeted residues in BoNT/B for mutagenesis studies are labeled in *green*. Syt II residues are marked in *purple*, with F54, F55, and I58 being highlighted. **b** A *schematic illustration* for construction of H_C_B mutant libraries and the BACTH assay. **c** H_C_B mutants identified in the BACTH assay were further analyzed by β-galactosidase activity assay, which reflects the levels of reconstituted adenylate cyclase (Student’s *t*-test, *n* = 6, *P < 0.05). *Error bars* represent SEM. **d** Point mutations at E1191, W1178, and S1199 were examined in pull-down assays, using immobilized m-Syt II (residues 1–87) or a mouse Syt II (1–87) containing F54L mutation that mimics h-Syt II sequence (designated as h-Syt II). Bound H_C_B variants were detected by immunoblot analysis detecting the HA-tag fused to H_C_B. Additional H_C_B mutations at other sites are shown in Supplementary Fig. 2. One of two independent experiments is shown

The co-crystal structure of BoNT/B bound to rodent Syt II has been determined^19, 20, 38^, providing a sound basis for us to select a total of 19 residues in BoNT/B that form the Syt II-binding pocket for mutagenesis studies (Fig. 1a and Supplementary Table 1). Our strategy was to saturate each of these 19 positions with all 20 possible amino acids, aiming to identify all single-residue mutations that increase binding to h-Syt II. To do so, we utilized the bacterial adenylate cyclase two-hybrid approach (BACTH)^39^. Briefly, the H_C_ of BoNT/B (H_C_B) was subcloned into a vector in frame with a split fragment (designated T18) of a bacterial adenylate cyclase. This T18-H_C_B fusion construct was amplified via polymerase chain reaction using primers harboring random tri-nucleotides (NNN) at the selected position, generating a pool of constructs encoding all 20 possible amino acids at the selected site (Fig. 1b). Those constructs were then co-transformed into bacteria (*Escerichia coli* strain BTH101) together with a construct that constitutively expresses the fragment of h-Syt II containing the toxin-binding site (residues 1–80) fused with the other half of the split bacterial adenylate cyclase (designated T25). Binding of H_C_B to h-Syt II brings T18 and T25 together and recovers the activity of adenylate cyclase, which leads to production of cAMP, expression of the *lacZ* gene, and results in blue colonies on X-Gal plates (Fig. 1b).

We used T18 paired with T25 as the negative control, which started to show weak blue colonies 72 h post plating; this was the background level. We used T18-H_C_B paired with T25 fused to mouse Syt II (m-Syt II, residues 1–87) as the positive control, which showed dark blue colonies after 48 h. Pairing T18-H_C_B with T25-h-Syt II resulted in light blue colonies after 68 h, suggesting that the BACTH assay is sensitive enough by 68 h to pick up even the weak interaction between H_C_B and h-Syt II. On the basis of these controls, we chose a rather conservative cutoff time of 64 h post plating to ensure that all promising point mutations were identified in our screen. Four locations showed blue colonies by 64 h, with dark blue colonies at E1191 (22.7% of the total number of colonies), and light blue colonies at W1178 (7.8%), Y1183 (4.0%), and S1199 (5.1%; Supplementary Table 2).

Plasmids were extracted and sequenced from these blue colonies, revealing the specific mutations at each site: E1191M/C/V/Q/L/Y, Y1183C/P, S1199W/E/Y/H, and W1178Y/Q. These mutations were then validated by measuring β-galactosidase activity in bacteria, which reflects the levels of reconstituted adenylate cyclase (Fig. 1c). Four mutations at E1191, E1191M/C/V/Q, showed stronger β-galactosidase activity than other mutations, suggesting that these four mutations resulted in strong binding to h-Syt II.

To further validate the results from the BACTH screen, we generated and examined a series of mutations at the E1191 site and a few other locations by pull-down assays. Glutathione-*S*-transferase (GST)-tagged m-Syt II fragment was used as a positive control. H-Syt I fragment was created by mutating F54 in mouse Syt II (1–87) to L, mimicking the human sequence (designated as h-Syt II). GST-tagged Syt II fragments were immobilized on beads and used to pull down H_C_B. Bound H_C_B was detected via an HA-tag fused to the N terminus of H_C_B by immunoblot analysis. E1191M/C/V/Q showed strong binding to h-Syt II, while E1191L/Y and S1199Y showed relatively weak binding to h-Syt II (Fig. 1d). These results are consistent with the β-galactosidase activity assay (Fig. 1c). W1178Y showed weak interactions with h-Syt II in the β-galactosidase activity assay, but did not show any detectable binding to h-Syt II in pull-down assays (Fig. 1d), suggesting that the pull-down assay is less sensitive in detecting weak interactions than the β-galactosidase activity assay or the BACTH screen. To confirm the specificity of the BACTH screen, we further evaluated a total of 18 point mutations at a few selected locations using pull-down assays, but none resulted in binding to h-Syt II (Supplementary Fig. 2).

### Combinational mutations in H_C_B

**Fig. 2:**
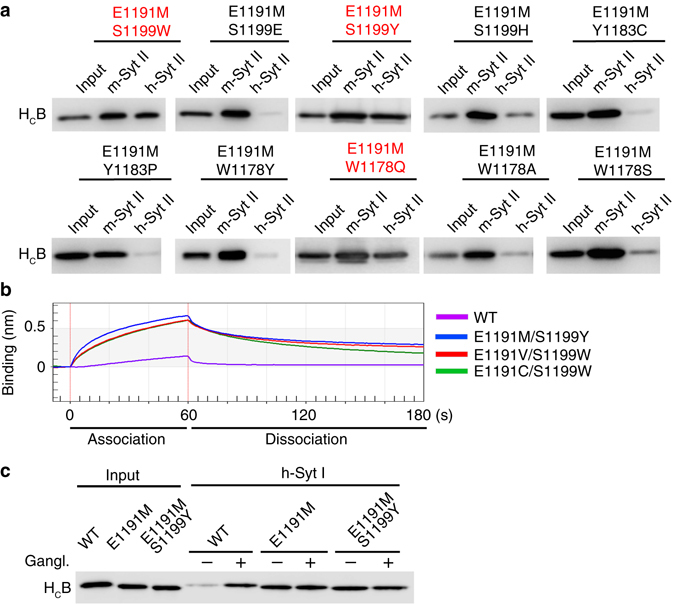
Combinational mutations in H_C_B enhance its binding to h-Syt II and Syt I. **a** H_C_B double mutations were examined in pull-down assays for their abilities to bind m-Syt II vs. h-Syt II. Three double mutations that showed robust binding to h-Syt II are marked in *red*. One of two independent experiments is shown. **b** Binding of H_C_B to h-Syt II was quantified by the BLI assay. Representative association and dissociation curves are shown for WT H_C_B and three H_C_B mutants: E1191M/S1199Y, E1191V/S1199W, and E1191C/S1199W. Binding parameters for all 12 double mutations are listed in Table 1, and their representative binding traces are shown in Supplementary Fig. 3. **c** Binding of WT H_C_B, H_C_B (E1191M), and H_C_B (E1191M/S1199Y) to immobilized GST-tagged h-Syt I (residues 1–80) was examined in pull-down assays, with (+) or without (−) gangliosides (Gangl.). Binding of WT H_C_B to h-Syt I requires the presence of gangliosides, while H_C_B (E1191M) and H_C_B (E1191M/S1199Y) bind to h-Syt I in the absence of gangliosides. One of two independent experiments is shown. Further analysis of H_C_B binding to h-Syt I by the BLI assay is shown in Supplementary Fig. 4 and Table 1

We next explored whether binding of H_C_B (E1191M/C/V/Q) to h-Syt II can be further enhanced by including a secondary mutation at a different location. Using E1191M as a primary mutation, we screened double mutations covering all eight identified mutations at the other three sites (W1178/Y/Q, Y1183/C/P, and S1199/W/E/Y/H), as well as two additional mutations at W1178 (W1178A/S). These 10 double mutants were analyzed for their ability to bind h-Syt II in pull-down assays (Fig. 2a). E1191M/Y1183C and E1191M/Y1183P reduced binding to h-Syt II compared to E1191M alone (Fig. 2a). This is likely because E1191 and Y1183 are spatially close (Fig. 1a) and the two mutations are not compatible with each other. Similarly, combining S1199E, S1199H, or W1178Y/A/S with E1191M resulted in less binding to h-Syt II than E1191M alone (Fig. 2a). Only three of the double mutations, E1191M/S1199W, E1191M/S1199Y, and E1191M/W1178Q, displayed robust binding to h-Syt II.

We then generated all 12 combinations between the primary mutations E1191M/C/V/Q and the three compatible secondary mutations S1199W/Y and W1178Q. In addition, we also generated a triple mutation E1191M/S1199W/W1178Q. The binding affinities (*K*_D_) of these mutants toward h-Syt II were measured using biolayer interferometry assay (BLI), with the parameters listed in Table 1 and representative traces shown in Supplementary Fig. 3. Briefly, GST-tagged Syt fragments were immobilized onto a probe, which was then exposed to purified H_C_B at different concentrations (association phase, Fig. 2b), followed by washing steps (dissociation phase, Fig. 2b). Binding of WT H_C_B to m-Syt II served as a positive control, which showed a *K*_D_ of 0.13 µM (Table 1). Binding of WT H_C_B to h-Syt II was too weak to be reliably determined in this assay, with an estimated *K*_D_ > 20 µM (Fig. 2b, Supplementary Fig. 3 and Table 1). The E1191M mutant yielded a *K*_D_ of 6.7 µM to h-Syt II. The majority of double mutants further improved the binding affinity, with *K*_D_ between 0.59 and 4.3 µM (Supplementary Fig. 3 and Table 1). The top two double mutants, E1191M/S1199Y (*K*_D_ = 0.72 µM) and E1191V/S1199Y (*K*_D_ = 0.59 µM), offered affinity an order of magnitude higher than the E1191M mutant (Table 1). The triple mutant E1191M/S1199W/W1178Q showed a binding affinity similar to the double mutant E1191M/S1199W, indicating that adding the third mutation site may not necessarily offer any further improvement (Table 1). We note that the *K*_D_ measured here reflects direct binding of BoNT/B to Syt II without the co-receptor gangliosides. BoNT/B would have much higher affinity toward neuronal surfaces by binding to both Syt II and gangliosides simultaneously.

**Table 1:** Interactions between GST-Syt II/Syt I and H_C_B variants measured by BLI

Ligand	H_C_B	*k*_on_ (M^−1^s^−1^)	*k*_on_ error	*k*_off_ (s^−1^)	*k*_off_ error	*K*_D_ (µM)
GST-m-Syt II	WT	1.47 × 10^4^	5.89 × 10^2^	1.98 × 10^−3^	5.18 × 10^−5^	0.13
GST-h-Syt II	WT	N/A	N/A	N/A	N/A	>20
	E1191M	5.21 × 10^3^	2.11 × 10^2^	3.51 × 10^−2^	4.62 × 10^−4^	6.7
	E1191M, S1199W	1.65 × 10^4^	3.87 × 10^2^	1.81 × 10^−2^	1.79 × 10^−4^	1.10
	E1191M, S1199Y	1.56 × 10^4^	3.72 × 10^2^	1.13 × 10^−2^	1.74 × 10^−4^	0.72
	E1191M, W1178Q	2.23 × 10^4^	1.33 × 10^3^	3.52 × 10^−2^	8.25 × 10^−4^	1.58
	E1191C, S1199W	1.32 × 10^4^	4.70 × 10^2^	1.19 × 10^−2^	8.90 × 10^−4^	0.90
	E1191C, S1199Y	1.47 × 10^4^	4.96 × 10^2^	1.44 × 10^−2^	1.91 × 10^−4^	0.98
	E1191C, W1178Q	6.89 × 10^3^	5.18 × 10^2^	2.96 × 10^−2^	3.23 × 10^−4^	4.30
	E1191Q, S1199W	8.28 × 10^3^	5.50 × 10^2^	2.21 × 10^−2^	3.34 × 10^−4^	2.67
	E1191Q, S1199Y	2.42 × 10^4^	1.16 × 10^3^	2.85 × 10^−2^	4.60 × 10^−4^	1.18
	E1191Q, W1178Q	N/A	N/A	N/A	N/A	>20
	E1191V, S1199W	1.86 × 10^4^	6.49 × 10^2^	1.30 × 10^−2^	2.44 × 10^−4^	0.70
	E1191V, S1199Y	2.31 × 10^4^	9.18 × 10^2^	1.35 × 10^−2^	2.68 × 10^−4^	0.59
	E1191V, W1178Q	6.08 × 10^3^	4.64 × 10^2^	1.83 × 10^−2^	2.75 × 10^−4^	3.01
	E1191M, S1199W, W1178Q	1.52 × 10^4^	3.49 × 10^2^	1.72 × 10^−2^	1.71 × 10^−4^	1.13
GST-h-Syt I	WT	N/A	N/A	N/A	N/A	>20
	E1191M, S1199Y	4.82 × 10^3^	2.96 × 10^2^	1.40 × 10^−2^	4.55 × 10^−4^	2.90
	E1191V, S1199Y	2.71 × 10^3^	9.11 × 10^1^	1.58 × 10^−2^	2.10 × 10^−4^	5.82

Binding parameters were calculated using the Blitz system software. Representative binding and dissociation curves are shown in Supplementary Figs. 3 and 4

### H_C_B mutants showed enhanced binding to h-Syt I

We next examined whether H_C_B mutants still bind to h-Syt I, which is highly homologous to Syt II and is also a functional receptor for BoNT/B (Supplementary Fig. 1). Binding of WT H_C_B to Syt I can be detected only in the presence of co-receptor gangliosides in pull-down assays, as Syt I has a lower binding affinity toward BoNT/B compared to Syt II^15, 30^. We found that both E1191M and E1191M/S1199Y mutants bound to h-Syt I in the absence of gangliosides in pull-down assays (Fig. 2c), suggesting that these mutants may enhance binding to h-Syt I. To confirm this finding, we analyzed binding of H_C_B containing E1191M/S1199Y (H_C_B_MY_) or E1191V/S1199Y (H_C_B_VY_) toward h-Syt I using the BLI assay (Supplementary Fig. 4 and Table 1). While binding of WT H_C_B to h-Syt I was too weak to be reliably measured, H_C_B_MY_ and H_C_B_VY_ showed *K*_D_ of 2.9 and 5.82 µM toward h-Syt I, respectively (Table 1). As H_C_B_MY_ showed a slightly higher binding affinity toward h-Syt I and a lower dissociation constant for both Syt I and Syt II compared to H_C_B_VY_, we selected this double mutant for further characterizations.

### H_C_B_MY_ binds to h-Syt II on neuronal surfaces

**Fig. 3:**
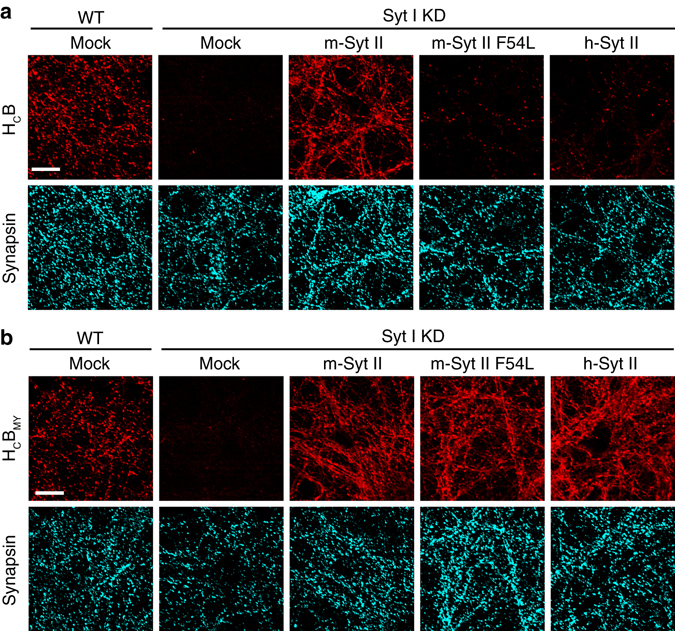
H_C_B_MY_ shows robust binding to neurons that express h-Syt II. **a** Humanized neurons were created by KD of endogenous Syt I and by expressing full-length h-Syt II in cultured rat cortical neurons via lentiviral transduction. KD efficiency was validated by immunoblot (Supplementary Fig. 5). Neurons that express full-length m-Syt II or m-Syt II (F54L) served as controls. These neurons were exposed to WT H_C_B (100 nM) followed by immunostaining analysis. Bound H_C_B was detected via an HA-tag fused to H_C_B. Synapsin was labeled as a marker for presynaptic terminals. WT H_C_B bound to WT neurons but not to Syt I KD neurons. Expression of full-length m-Syt II restored binding of H_C_B. Expression of full-length m-Syt II (F54L) or full-length h-Syt II resulted in only low levels of binding of WT H_C_B compared to m-Syt II. **b** Syt I KD abolished binding of H_C_B_MY_ (100 nM) to neurons. The binding was rescued by expression of full-length m-Syt II, m-Syt II (F54L), or h-Syt II. *Scale bars* represent 20 µm. One of two independent experiments is shown

We next examined whether the H_C_B_MY_ mutant binds to h-Syt II on physiologically relevant neuronal surfaces. As a model, we utilized cultured rat cortical neurons, which express Syt I but not Syt II^13^. Thus, knocking down (KD) Syt I leaves these neurons with no endogenous receptors. Expressing full-length h-Syt II in these Syt I KD neurons then creates “humanized” neurons with only h-Syt II as the toxin receptor (Supplementary Fig. 5)^18^. WT H_C_B bound strongly to rat neurons, mediated by Syt I in the presence of gangliosides. As expected, the binding was largely abolished after KD endogenous Syt I (Fig. 3a). Expression of exogenous full-length m-Syt II restored binding of WT H_C_B. Expression of exogenous h-Syt II, or a m-Syt II containing F54L mutation, resulted in much lower levels of binding of WT H_C_B compared to m-Syt II (Fig. 3a). In contrast, H_C_B_MY_ showed robust binding to neurons that express m-Syt II, h-Syt II, or m-Syt II (F54L), demonstrating that H_C_B_MY_ has an enhanced ability to bind h-Syt II on neuronal surfaces (Fig. 3b).

### BoNT/B mutant showed enhanced efficacy in humanized neurons

A key question is whether enhanced binding to receptors improves functional efficacy, i.e., blocking neurotransmission in neurons. To test this, we produced full-length WT BoNT/B and E1191M/S1199Y double-mutant toxin (BoNT/B_MY_) recombinantly in *E. coli* (Supplementary Fig. 6a). BoNT/B_MY_ entered cultured rat cortical neurons and cleaved its substrate VAMP2, as detected by immunoblot analysis (Supplementary Fig. 6b). BoNT/B_MY_ activity is readily neutralized by both a rabbit polyclonal anti-BoNT/B antibody and trivalent horse antisera (Supplementary Fig. 6b), confirming that E1191M/S1199Y mutations do not change the ability of currently available antisera to neutralize the toxin.

**Fig. 4:**
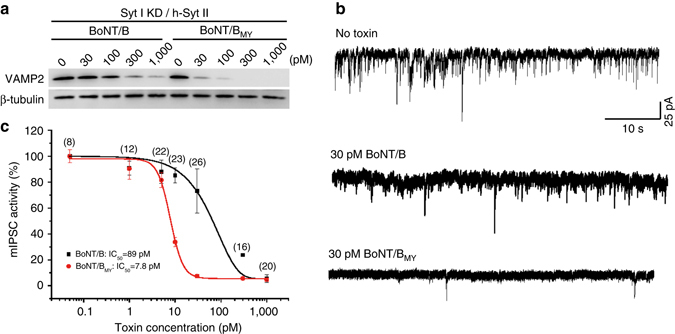
BoNT/B_MY_ shows enhanced functional efficacy in neurons that express h-Syt II. **a** Humanized neurons were created as described in Fig. 3a. Neurons were exposed to a gradient of full-length WT BoNT/B or BoNT/B_MY_ for 24 h. Cell lysates were harvested and subjected to immunoblot analysis. β-tubulin served as an internal loading control. BoNT/B_MY_ cleaved more VAMP2 than WT BoNT/B at all concentrations tested, indicating that BoNT/B_MY_ entered neurons more efficiently than WT BoNT/B. One of three independent experiments is shown. **b**,**c** Humanized neurons were exposed to a gradient of WT BoNT/B or BoNT/B_MY_ for 24 h. The mIPSC was recorded by a whole-cell patch-clamp approach. **b** Representative mIPSC recordings at 30 pM toxins. **c** The mIPSC activities vs. toxin concentrations, normalized to neurons that were not exposed to toxins. The numbers of recorded neurons for each data point are noted within *parentheses*. The same number of neurons was recorded for WT BoNT/B and BoNT/B_MY_. The half maximum inhibitory concentration (IC_50_) was determined to be 89 pM for WT BoNT/B and 7.8 pM for BoNT/B_MY_, demonstrating that enhancing binding to receptors increased functional efficacy of toxins in neurons. Statistical analysis was performed with Student’s *t*-test (*P < 0.01). All data shown are means ± SEM

We then compared the activity of BoNT/B_MY_ vs. WT BoNT/B on humanized neurons. As shown in Fig. 4a, more VAMP2 was cleaved in neurons exposed to BoNT/B_MY_ compared to neurons exposed to BoNT/B at each toxin concentration tested, indicating that BoNT/B_MY_ targeted and entered neurons more efficiently than WT toxin. We next monitored neurotransmitter release directly by recording miniature inhibitory postsynaptic currents (mIPSCs) using whole-cell patch-clamp. The frequency of mIPSCs reflects the activity of neurotransmitter release in a population of neurons. Entry of BoNT/B into presynaptic terminals blocks release of neurotransmitter, thus reducing frequency of mIPSCs (Fig. 4b). Humanized neurons were exposed to a gradient of WT BoNT/B or BoNT/B_MY_. As shown in Fig. 4c, BoNT/B_MY_ showed a greatly enhanced potency, with a half maximum inhibitory concentration (IC_50_) ~11-fold lower than WT toxin. These data demonstrate that enhanced binding to receptors increased the functional efficacy of toxins in neurons.

### Binding of BoNT/B4 to h-Syt II

**Fig. 5:**
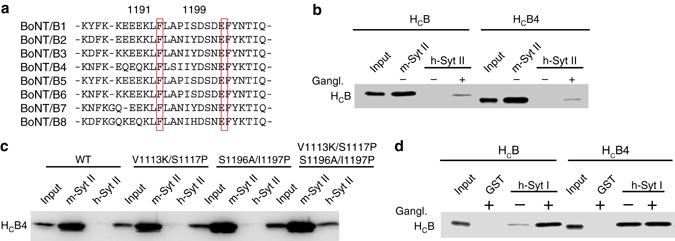
Variations in BoNT/B subtypes influence their binding to h-Syt II and h-Syt I. **a** Sequence alignment of BoNT/B subtypes in the region around residues E1191/S1199. **b** H_C_B4 showed robust binding to m-Syt II, but no detectable binding to h-Syt II without gangliosides, which is similar to H_C_B. Both showed low levels of binding to h-Syt II in the presence of gangliosides. **c** There are four residues that differ between H_C_B4 and H_C_B within the 19 key residues in the Syt-binding pocket besides E1191Q/S1199Y. Replacing all four residues in H_C_B4 with the corresponding residues in H_C_B created a mutant H_C_B4 (V1113K/S1117P/S1196A/I1197P) that can bind to h-Syt II. The sequence alignment between H_C_B and H_C_B4 is shown in Supplementary Fig. 7. **d** Binding of H_C_B and H_C_B4 to immobilized GST-tagged h-Syt I was examined in pull-down assays. Binding of H_C_B to h-Syt I requires the presence of gangliosides, while H_C_B4 is capable of binding to h-Syt I without gangliosides in pull-down assays

Finally, we explored whether sequence variations in BoNT/B subtypes may affect their ability to bind to h-Syt II. BoNT/B has eight subtypes known to date (BoNT/B1–B8), with up to 7% sequence variation^40^. Sequence alignment revealed variations at both E1191 and S1199 (Fig. 5a). Intriguingly, there are cases of E1191Q (BoNT/B4, B8) and S1199Y (BoNT/B2, B3, B4, B7). There is even a combination of E1191Q and S1199Y (BoNT/B4)—such a double mutation in BoNT/B resulted in robust binding to h-Syt II (Fig. 2a and Table 1). However, H_C_B4 is similar to WT H_C_B: neither pulled down h-Syt II without gangliosides and only bound to h-Syt II weakly in the presence of gangliosides (Fig. 5b). This is likely due to other residue changes in H_C_B4 from H_C_B. Among the 19 key residues at the BoNT/B-Syt interface, there are four other residues that differ between BoNT/B and B4 besides E1191Q/S1199Y (Supplementary Fig. 7). Replacing all four residues in H_C_B4 with the corresponding residues in BoNT/B increased its binding to h-Syt II (Fig. 5c), although not to the same level as H_C_B_MY_, suggesting that residues other than the 19 selected ones may also influence the binding affinity. Interestingly, H_C_B4 showed robust binding to h-Syt I in the absence of gangliosides, suggesting that this subtype has a better binding affinity toward Syt I than BoNT/B (Fig. 5d).

## Discussion

By combining structure-based rational design and BACTH-based saturation mutagenesis, we identified a series of point mutations in BoNT/B that improve its binding to h-Syt II. These point mutations were further examined in combinations, identifying several double mutations that display much higher binding affinity toward h-Syt II than WT BoNT/B. Furthermore, mutated BoNT/B_MY_ showed ~11-fold higher efficacy than WT BoNT/B in neurons that express h-Syt II, demonstrating that enhanced binding to toxin receptors translates to a higher functional efficacy in neurons.

Our findings indicate that E1191 is located at a key position modulating the binding affinity between BoNT/B and Syt I/II. It has been previously reported that E1191L increases binding of BoNT/B to Syt I^41^, likely due to removing a potentially unfavorable negative charge. Consistently, E1191L/Y also increased binding to h-Syt II. However, E1191M/C/V/Q showed much higher levels of binding to h-Syt II than E1191L/Y, suggesting that M/C/V/Q may favor new side-chain to side-chain interactions with nearby residues in h-Syt II, in addition to removing the negative charge at E1191. The nature of new side-chain interactions remains unknown, and solving the co-crystal structures of BoNT/B_E1191M/C/V/Q_ in complex with h-Syt II will be essential to provide a molecular understanding.

The BACTH screen also identified W1178Y/Q, Y1183C/P, and S1199W/E/Y/H as mutations that increase binding to h-Syt II. S1199Y has previously been reported to increase binding of BoNT/B to Syt II, possibly as a result of a new π-stacking interaction between Y1199 and F47 of Syt II^19^. How other mutations modulate the binding affinity remains unknown. Intriguingly, residues at 1191 and 1199 sites vary across different BoNT/B subtypes. For instance, the 1191 site can be E (B1, B3, B5/6/7), K (B2), or Q (B4, B8), and the 1199 site can be S (B1, B5, B6), Y (B2/3/4, B7), or H (B8). Such residue changes may alter the binding affinity toward Syt I/II. For instance, we found that BoNT/B4 binds better to h-Syt I than BoNT/B1, likely because it contains Q at the 1191 site and Y at the 1199 site. It appears that nature has already sampled a repertoire of residues for their ability to alter toxin–receptor interactions at a few key locations, which were also identified in our BACTH screen. With a growing number of subtype toxins identified, their sequence variations may provide a rich resource for mining residues that could modulate toxin–receptor interactions.

We limited our screen to the 19 major residues within the Syt II-binding pocket in BoNT/B. It remains possible that additional mutations at residues beyond these 19 may influence/enhance binding to h-Syt II, considering that a mutated BoNT/B4 containing the same set of residues as BoNT/B1 at these 19 locations still binds to h-Syt II poorly compared to BoNT/B_MY_. Methods such as directed evolution and computational approaches might be utilized to identify additional mutations that further enhance the binding affinity. However, the benefit of introducing additional mutations might be limited. As the presence of gangliosides greatly enhances the overall binding affinity toward neuronal surfaces, it might be less meaningful to further increase toxin–Syt interactions beyond what has been achieved with double mutations such as E1191M/S1199Y.

Both Syt I and Syt II are functional receptors for BoNT/B, which recognizes a short conserved region in Syt I/II adjacent to their transmembrane domains. Structural modeling showed that the side chains that interact with BoNT/B are highly similar between Syt I and Syt II, which may explain why double mutations such as E1191M/S1199Y increased binding to both Syt I and Syt II (Supplementary Fig. 1a). h-Syt I contains the same sequence within the toxin-binding region as rat/mouse Syt I, with only a single residue difference (Q in r/m-Syt I, E in h-Syt I at position 45, Supplementary Fig. 1b). This residue is located away from the BoNT/B-binding interface. Thus, there is no structural difference in the binding to BoNT/B between h-Syt I and rat/mouse Syt I.

A major difference between m/h-Syt I and h-Syt II is that m/h-Syt I binds strongly to BoNT/B in the presence of gangliosides, whereas h-Syt II showed only weak binding to BoNT/B in the presence of gangliosides because of the F-to-L change (Figs. 2c and 5b). Consistently, H_C_B and H_C_B_MY_ showed similar levels of binding to rat neurons that express endogenous Syt I (Fig. 3). This is likely because Syt I was able to mediate robust binding of H_C_B in the presence of gangliosides; thus any differences on Syt I binding between H_C_B and H_C_B_MY_ are masked on neuronal surfaces. In contrast, H_C_B showed only weak binding to neurons that express h-Syt II, indicating that the defect in binding to h-Syt II cannot be compensated for by the presence of gangliosides (Fig. 3a). In this case, mutating H_C_B to enhance its binding to h-Syt II is required to achieve robust binding to neurons that express h-Syt II. Considering that Syt II appears to be the dominant form expressed in the motor nerve terminal^31^, increased binding to h-Syt II is likely the dominant factor in any improvement of efficacy for BoNT/B_MY_ in human neurons. We utilized humanized neurons that overexpress h-Syt II, which provided proof-of-principle evidence, suggesting that BoNT/B_MY_ has a higher potency than WT BoNT/B in humans. The efficacy of BoNT/B_MY_ on human motor neurons and physiologically relevant human tissues remains to be established.

BoNT/DC and BoNT/G recognize surface residues on Syt I/II similar to those recognized by BoNT/B, and both showed diminished binding to h-Syt II^18^. The co-crystal structures of BoNT/DC in complex with Syt I/II have been solved^42^; therefore, a similar BACTH screen, targeting key residues in the binding interface, may identify mutations in BoNT/DC that increase its affinity to h-Syt II. It is intriguing to note that humans are known to be less sensitive than mice to BoNT/D because of a single residue difference in human VAMP1, from M48 (mouse VAMP1 sequence number) in mice to I48 in humans. BoNT/D showed significantly reduced cleavage efficiency for human VAMP1 compared to mouse VAMP1^43–45^. Considering that BoNT/DC shares the same protease domain as BoNT/D, humans possess residue changes in both a toxin receptor and a toxin substrate that render humans less sensitive to BoNT/DC. Although we cannot exclude the possibility that these two changes are random events, such a co-incidence strongly suggests that BoNTs have exhibited a selective pressure on humans^43^.

Medical use of BoNTs is a remarkable example of transforming a deadly toxin into an effective therapeutic. With the rapid expansion of their medical applications, there is a growing interest in engineering BoNTs to improve their therapeutic efficacy and reduce their adverse effects^46–53^. The BoNT/B mutants identified here have the potential to facilitate development of a new generation of therapeutic toxins with improved efficacy in patients. In addition, such modified BoNT/B and its H_C_ will also become valuable scientific tools and delivery vehicles for targeting human neurons.

## Methods

### Materials and constructs

The following antibodies were purchased from the indicated vendors: Synapsin I (Clone 46.1, Synaptic Systems), VAMP2 (Clone 69.1, Synaptic Systems), HA (16B12, Covance), Syt I (Clone mAB48, The Developmental Studies Hybridoma Bank), Syt II (Clone 26, BD Bioscience), polyclonal rabbit anti-BoNT/B (Metabiologics), and β-tubulin III (ab18207, Abcam). Equine polyclonal antisera against BoNT/A/B/E were generously provided by S. Sharma (FDA). Bovine mixed brain gangliosides were purchased from Matreya LLC (Pleasant Gap, PA) and were reconstituted in Tris-buffered saline (TBS: 20 mM Tris, 150 mM NaCl) as previously described^14^. The cDNAs encoding H_C_B (residue 857–1291, Genbank: ACA46990.1) and H_C_B4 (Genbank: EF051570) were codon-optimized for *E. coli* expression and synthesized by GenScript Inc. (New Brunswick, NJ). The following cDNAs were generously provided by the indicated groups: rat Syt I and mouse Syt II (E. Chapman, Madison, WI, USA), h-Syt I (R.B. Sutton, Lubbock, TX, USA). DNA encoding H_C_B was subcloned into pET28a vector, with both a His6 tag and an HA tag (YPYDVPDYA) fused to its N terminus. Mutations in H_C_B were generated using a Site-directed Mutagenesis Kit (Agilent Technologies, CA). GST-tagged Syt I/II fragments and Syt II F54L mutant were described previously^15, 18, 23^.

### Production of full-length BoNT/B and BoNT/B_MY_

The cDNA encoding full-length inactive BoNT/B_R370A,Y373F_ was codon-optimized for expression in *E. coli*. It was cloned into pET32a via NdeI/BamHI to add a His6 tag to the C terminus. The construct was reverted back to its active WT form by site-directed mutagenesis (Agilent Technologies). E1191M/S1199Y mutations were introduced to produce the BoNT/B_MY_ double mutant. The constructs were transformed into *E. coli* (strain BL21 DE3), grown in mTB + 100 μg ml^−1^ ampicillin, and induced with 1 mM IPTG at 16 °C for 20 h. The bacteria were harvested and lysed by ultrasonication in 5 ml of 0.5 M NaCl in 50 mM Tris pH 8 and 0.5 μL Benzonase, per gram of pellets. The crude lysate was clarified by centrifugation at 4000 g for 1 h, and BoNT/B was captured on a HisTrap HP column (GE) and eluted with 0.1 M imidazole (0.5 M NaCl, 50 mM Tris, pH 8) by FPLC (GE). The eluate was desalted into 125 mM NaCl in 50 mM Tris pH 8 using a HiPrep 26/10 desalting column (GE), and then concentrated to 0.6 mg ml^−1^ using a 10 kDa MWCO spin filter. The concentrate was treated with 0.1 μg ml^−1^ endoproteinase Lys-C for 2 h at 37 °C and then adjusted to 1 M (NH_4_)_2_SO_4_ before final purification by hydrophobic interaction chromatography with phenyl Sepharose HP (GE). Proteins were eluted with a 1–0 M (NH_4_)_2_SO_4_ in 50 mM Tris pH 8 linear gradient. The fractions containing the di-chain BoNT/B were pooled, desalted, concentrated, and stored at −80 °C.

### BACTH assay

The BACTH assay was performed according to the manufacturer’s instructions (Euromedex). Two compatible plasmids, pUT18C and pKT25, were selected for the screen. H-Syt II luminal domain (residues 1–80) was cloned into pKT25 to generate pKT25-h-Syt II. H_C_B was cloned in pUT18C for producing T18-H_C_B. H_C_B mutant libraries were created with primers containing random nucleotide triplets (NNN) at designated positions. Each library was co-transformed with the pKT25-h-Syt II plasmid into *E. coli* indicator strain BTH101 by electroporation and screened on LB agar plates containing 100 µg ml^−1^ Ampicillin, 50 µg ml^−1^ Kanamycin, 0.5 mM IPTG, and 40 µg ml^−1^ X-Gal. The experimental plates were incubated at 30 °C for 64 h. Plasmids were extracted from blue colonies and sequenced. The total colony number determined the possibility of covering all 20 amino acids at the selected mutation site. This was calculated by Clark-Carbon equation: *P* = 1−(1−*f*)^*N*^, where *f* reflects the number of possible residues (*f* = 1/20 here, as there are 20 different amino acids), and *N* is the total number of colonies. With a minimal number of 380 colonies in our assays, the probability of covering all 20 amino acids at each position is >99.8%.

### β-galactosidase activity assay

The assay was performed as previously described^54^. Briefly, *E. coli* BTH101 cells with plasmids of interest were inoculated into LB medium containing antibiotics and IPTG (0.5 mM). The culture was grown overnight at 37 °C to reach the stationary phase. The OD_600_ of the culture was recorded before harvesting. The culture was centrifuged, and cell pellets were washed twice with PBS and re-suspended in Z buffer (60 mM Na_2_HPO_4_, 40 mM Na_2_HPO_4_, 10 mM KCl, 1 mM MgSO_4_, and 20 mM DTT). Chloroform (1:10 (v/v)) and 0.1% SDS (1:20 (v/v)) were added to lyse cells. The cell lysates were then mixed with o-nitrophenyl-p-galactoside (4 mg ml^−1^ in Z buffer) at a 5:1 ratio and incubated at 28 °C for 10 min. The reaction was stopped by adding 80 µl of 1 M Na_2_CO_3_. β-galactosidase activity was calculated as *A*_420_/OD_600_.

### GST pull-down assays

Two types of pull-down assays were carried out. The first series was used to quickly screen binding of mutant H_C_B to GST-tagged m-Syt II (residues 1–87) and a mutant m-Syt II (F54L) that mimics h-Syt II. Briefly, 6 ml of *E. coli* expressing H_C_B were pelleted, re-suspended in 800 µl TBS, sonicated, and then incubated with 2% Triton X-100 for 1 h at 4 °C. Samples were then spun down for 15 min in a microcentrifuge at 4 °C. The supernatants were collected and used for pull-down assays by incubation with 10 µg of GST–Syt proteins immobilized on glutathione-Sepharose beads (GE) at 4 °C for 1 h. Samples were washed three times in washing buffer (TBS with 0.5% Triton X-100) and analyzed by immunoblot analysis using anti-HA antibody. For mutants that showed enhanced binding to h-Syt II, further characterization was carried out by purifying these H_C_B mutants as His6-tagged proteins. Pull-down assays were then carried out using purified H_C_B (100 nM) and immobilized GST-Syt II in 100 µl TBS buffer plus 0.5% Triton X-100, with or without gangliosides (60 µg ml^−1^), for 1 h at 4 °C. Beads were washed three times using TBS buffer plus 0.5% Triton X-100. Ten percent of bound materials were subjected to SDS-PAGE followed by immunoblot analysis.

### Biolayer interferometry assay

The binding affinity between H_C_B variants and Syt I/Syt II was measured by BLI assay with the Blitz system (ForteBio, Fremont, CA). Briefly, the GST-tagged Syt I or Syt II (20 μg ml^−1^) was immobilized onto Dip and Read Anti-GST Biosensors (ForteBio) and balanced with PBS buffer. The biosensors were then exposed to series concentrations of H_C_B, followed by washing with PBS. Binding affinities (*K*_D_) were calculated using the Blitz system software following the manufacturer’s instructions (ForteBio).

### Neuron culture, lentivirus, and toxin binding/entry assay

All procedures were conducted in accordance with the guidelines approved by the Institute Animal Care and Use Committee (IACUC) at Boston Children’s Hospital (#3030). Rat cortical neurons were prepared from E18–19 embryos (from timed-pregnant rat, Sprague–Dawley strain, purchased from Charles River) as described previously^14^. Constructs for Syt I KD, m-Syt II, and h-Syt II expression in neurons were previously described^18^. Lentiviruses were added to neuron cultures at DIV5 (days in vitro), and toxin-binding/entry experiments were carried out on DIV12–14. Toxins were diluted in high-K^+^ buffer (87 mM NaCl, 56 mM KCl, 1.5 mM KH_2_PO_4_, 8 mM Na_2_HPO_4_, 0.5 mM MgCl_2_, and 1 mM CaCl) and pre-warmed to 37 °C. Neurons were exposed to the above toxin-containing buffers for 5 min at 37 °C, followed by washing with PBS. These neurons were either subjected to immunostaining analysis or incubated in toxin-free medium for an additional 24 h, followed by immunoblot analysis.

### mIPSC recording

Whole-cell patch-clamp recordings were made from cultured cortical neurons (DIV 14–18). The pipette solution contained (in mM): 135 CsCl, 10 HEPES, 1 EGTA, 1 Na-GTP, 4 Mg-ATP, and 10 QX-314 (pH 7.4, adjusted with CsOH). The resistance of pipettes filled with intracellular solution varied between 4 and 5 MΩ. After formation of the whole-cell configuration and equilibration of the intracellular pipette solution, the series resistance was adjusted to 10 MΩ. Synaptic currents were monitored with an EPC-10/2 amplifier (HEKA) at −70 mV holding potential. The bath solution contained (in mM): 140 NaCl, 5 KCl, 2 CaCl, 1 MgCl_2_, 10 HEPES, 10 glucose (pH 7.4, adjusted with NaOH). Spontaneous inhibitory postsynaptic currents and evoked inhibitory postsynaptic currents were pharmacologically inhibited by adding AMPA and NMDA receptor blockers CNQX and APV to the extracellular bath solution. Spontaneous mIPSCs were monitored in the presence of tetrodotoxin to block action potentials. Data were analyzed using Clampfit 10 (Molecular Devices), Origin8 software (Mocrocal Inc.), MiniAnalysis software (Synaptosoft), and Igor (Wavemetrics). Statistical analysis was performed with Student’s *t*-test (*P < 0.01). All data shown are means ± SEM.

### Data availability

The data and materials that support the findings of this study are available from the corresponding authors upon request.

## Electronic supplementary material

Supplementary Information
