# Supplementary material for: Engineered botulinum neurotoxin B with improved efficacy for targeting human receptors
Source: Nat Commun. 2017 Jul 3;8:53. doi: 10.1038/s41467-017-00064-y (PMC5495808; doi:10.1038/s41467-017-00064-y)
Supplement: Supplementary file 1 — Supplementary Information [file 41467_2017_64_MOESM1_ESM.pdf]

File Name: Supplementary Information
Description: Supplementary Figures and Supplementary Tables

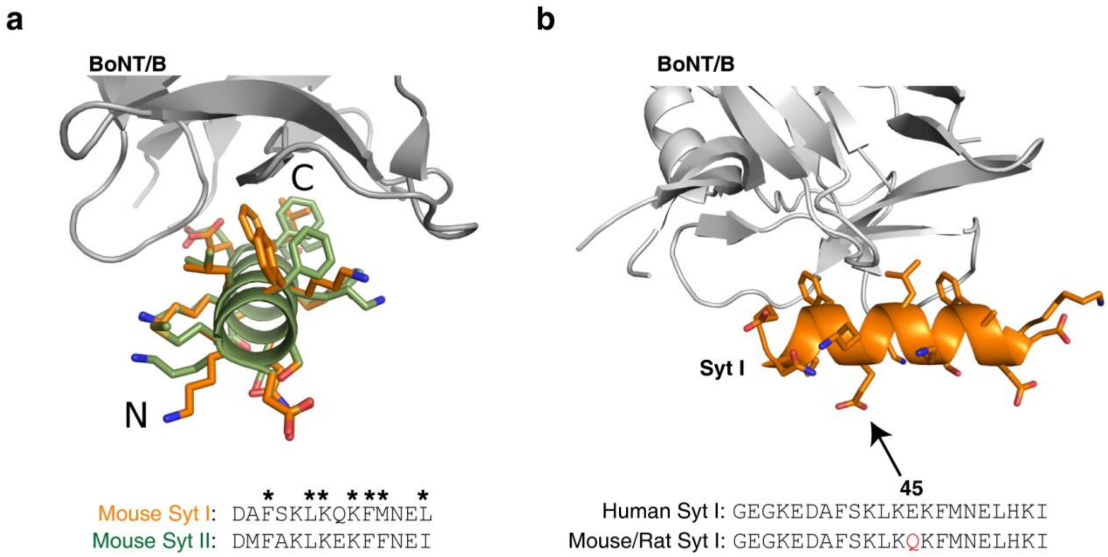

Supplementary Figure 1. Comparisons between Syt I and Syt II and between human and mouse Syt I at the BoNT/B-binding interface.

(**a**) Sequence alignment and structural comparison between mouse Syt I (orange) and Syt II (green). The asterisks highlights the conserved residues that interact with BoNT/B, which can be seen in the modelled structure of BoNT/B (gray) bound to Syt I (orange) and Syt II (green). Binding of Syt I (orange) to BoNT/B (gray) was modeled based on available BoNT/B-Syt II crystal structures (PDB: 4KBB).

(**b**) Sequence alignment of human Syt I versus mouse/rat Syt I within the BoNT/B-binding domain. The only difference, E45 in human Syt I and Q in mouse/rat-Syt I, is highlighted. The model shows that the side-chain of residue 45 (indicated by the arrow) is pointing outwards from the binding site, and does not interact with BoNT/B.

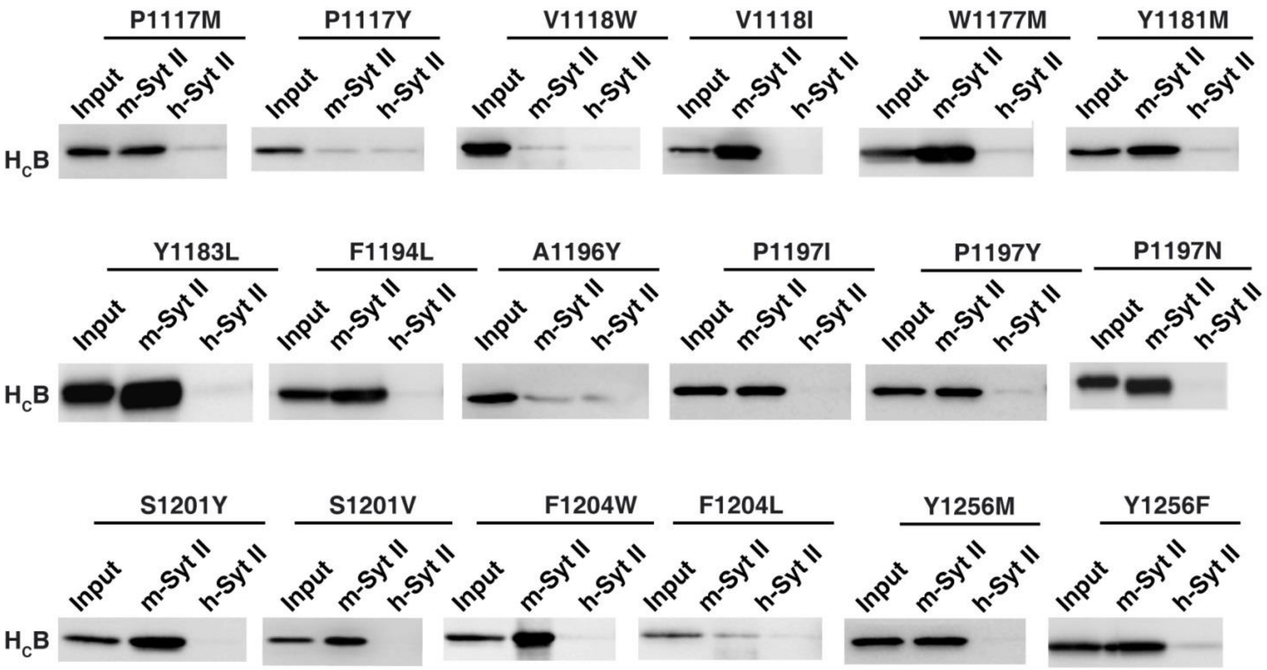

Supplementary Figure 2. Screening point mutations in H$_C$B for their ability to bind h-Syt II in pull-down assays.

Binding of the indicated H$_C$B mutants to m-Syt II and h-Syt II was examined in pull-down assays as described in Fig. 1e. None of the mutations tested showed detectable binding to h-Syt II.

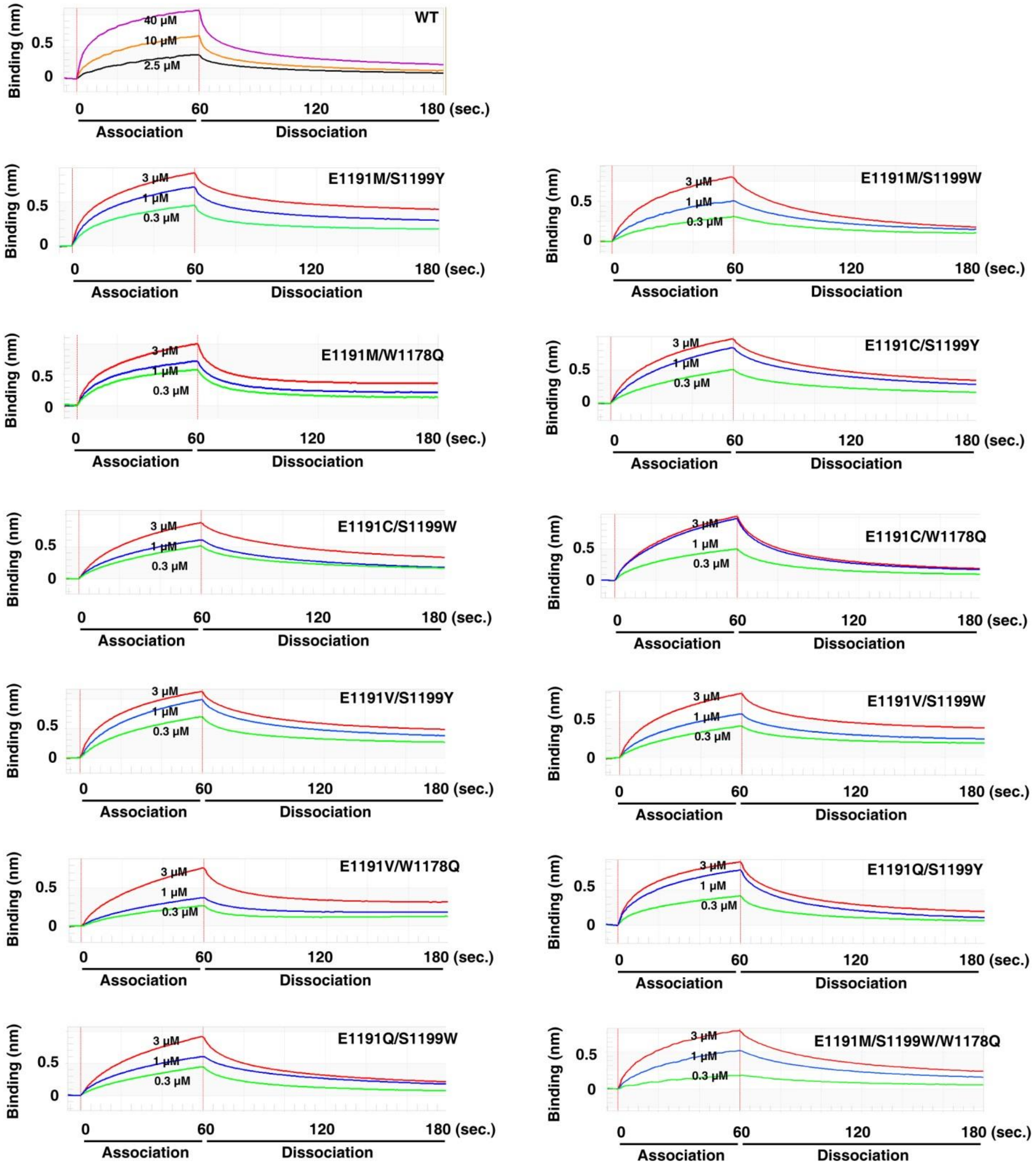

Supplementary Figure 3. Characterizing H_CB binding to h-Syt II using the BLI assay.

Experiments were carried out as described in Fig. 2b for the indicated WT and mutant H_CB using the BLI assay. The sensors containing immobilized h-Syt II were exposed to the indicated three concentrations of H_CB to determine the binding kinetics. Representative binding and dissociation curves are presented here. The binding parameters are shown in Table 1.

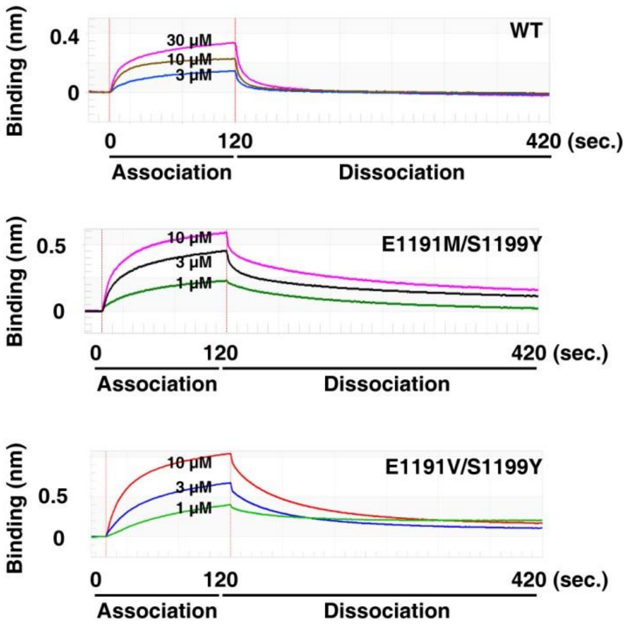

Supplementary Figure 4. Characterizing H_CB binding to h-Syt I using the BLI assay. Experiments were carried out for the indicated WT and mutant H_CB as described in Supplementary Fig. 3, except using immobilized h-Syt I on the BLI sensor. The representative binding traces are shown here. The binding parameters are listed in Table 1.

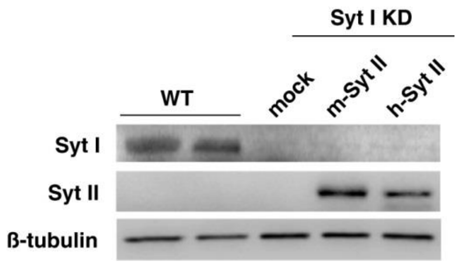

Supplementary Figure 5. Knocking down endogenous Syt I and expression of exogenous Syt II in rat cortical neurons.

Rat cortical neurons were prepared from E18–19 embryos. Lentiviruses for Syt I KD, m-Syt II, and h-Syt II expression were added to neuron cultures on DIV5. Neuron lysates were harvested on DIV12 for immunoblot analysis. β-tubulin served as an internal loading control. KD efficiency is high as Syt I was not detected. M-Syt II and h-Syt II showed similar levels of expression in neurons.

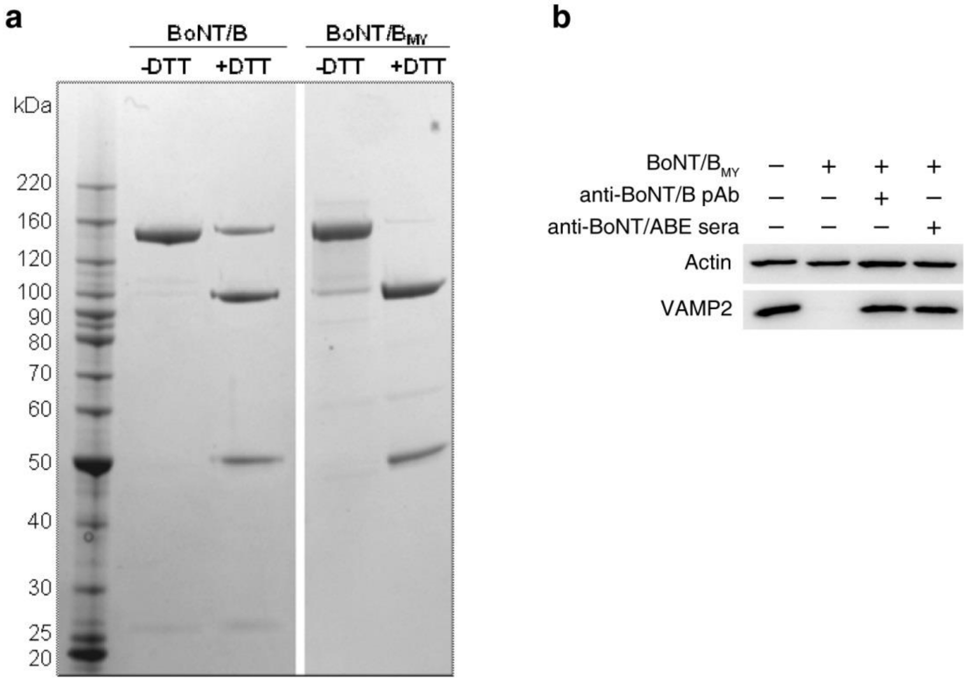

Supplementary Figure 6. BoNT/B$_{MY}$ produced in *E. coli* is active and can be neutralized by BoNT/B antibodies.

(**a**) Full-length BoNT/B and BoNT/B$_{MY}$ were purified from *E.coli* as described in the Method section. These toxins were activated by the endoproteinase Lys-C. SDS-PAGE analysis revealed that that WT BoNT/B toxin is ~93% pure with ~80% to be in its di-chain form, and BoNT/B$_{MY}$ toxin is ~86% pure with ~99% in its di-chain form.

(**b**) Cultured rat cortical neurons were exposed to BoNT/B$_{MY}$ (1 nM in culture medium, 16 h) that was pre-incubated with either rabbit poly-clonal anti-BoNT/B antibody (1:200 dilution in culture medium) or a trivalent horse antisera against BoNT/A, B, and E (1:40 dilution in culture medium). Neuron lysates were harvested 16 h later. Cleavage of VAMP2 was analyzed by immunoblot assays. BoNT/B$_{MY}$ is active as it cleaved VAMP2 in neurons. Both rabbit poly-clonal anti-BoNT/B antibody and trivalent horse antisera readily neutralized BoNT/B$_{MY}$. Actin served as an internal loading control. These results confirmed that E1191M/S1199Y mutations do not change the immunogenicity of BoNT/B.

```
BoNT/B1 (858)  SEILNNIILNLRYKDNNLIDLSGYGAKVEVYDGVELNDKNQFKLTSSANSKIRVTQN
BoNT/B4 (858)  SEILNNIILNLRYRDNNLIDLSGYGAKVEVYDGVKLNDKNQFKLTSSADSKIRVTQN

BoNT/B1 (915)  QNIIFNSVFLDFSVSFWIRIPKYKNDGIQNYIHNEYTIINCMKNNSGWKISIRGNRI
BoNT/B4 (915)  QNIIFNSMFLDFSVSFWIRIPKYRNDDIQNYIHNEYTIINCMKNNSGWKISIRGNRI

BoNT/B1 (972)  IWTLIDINGKTKSVFFEYNIREDISEYINRWFFVTITNNLNNAKIYINGKLESNTDI
BoNT/B4 (972)  IWTLIDINGKTKSVFFEYNIREDISEYINRWFFVTITNNLDNAKIYINGTLESNMDI

BoNT/B1 (1029) KDIREVIANGEIIFKLDGDIDRTQFIWMKYFSIFNTELSQSNIEERYKIQSYSEYLK
BoNT/B4 (1029) KDIGEVIVNGEITFKLDGDVDRTQFIWMKYFSIFNTQLNQSNIKEIYKIQSYSEYLK

                                          * ****
BoNT/B1 (1086) DFWGNPLMYNKEYYMFNAGNKNSYIKLKKDSPVGEILTRSKYNQNSKYINYRDLYIG
BoNT/B4 (1086) DFWGNPLMYNKEYYMFNAGNKNSYIKLVKDSSVGEILIRSKYNQNSNYINYRNLYIG
                                          ↑   ↑

                                                  *  * *       **  *** *
BoNT/B1 (1143) EKFIIRRKSNSQSINDDIVRKEDYIYLDFFNLNQEWRVYTYKYFKKEEEKLFLAPIS
BoNT/B4 (1143) EKFIIRRKSNSQSINDDIVRKEDYIHLDFVNSNEEWRVYAYKNFKEQEQKLFLSIIY
                                                                    ↑↑

                * **                                        *          *
BoNT/B1 (1200) DSDEFYNTIQIKEYDEQPTYSCQLLFKKDEESTDEIGLIGIHRFYESGIVFEEYKDY
BoNT/B4 (1200) DSNEFYKTIQIKEYDEQPTYSCQLLFKKDEESTDDIGLIGIHRFYESGVLRKKYKDY

BoNT/B1 (1257) FCISKWYLKEVKRKPYNLKLGCNWQFIPKDEGWTE
BoNT/B4 (1257) FCISKWYLKEVKRKPYKSNLGCNWQFIPKDEGWTE
```

Supplementary Figure 7. Sequence alignment between H_CB and H_CB4, with distinct residues marked in red.

The 19 key residues forming the Syt II-binding pocket are marked with asterisks. Within these 19 residues, six residues are different between H_CB and H_CB4, including E1191S and S1199Y. The other four different residues are marked with arrows and subjected to mutagenesis studies as described in Fig. 5c.

Figure 1d & Supplementary Fig. 2

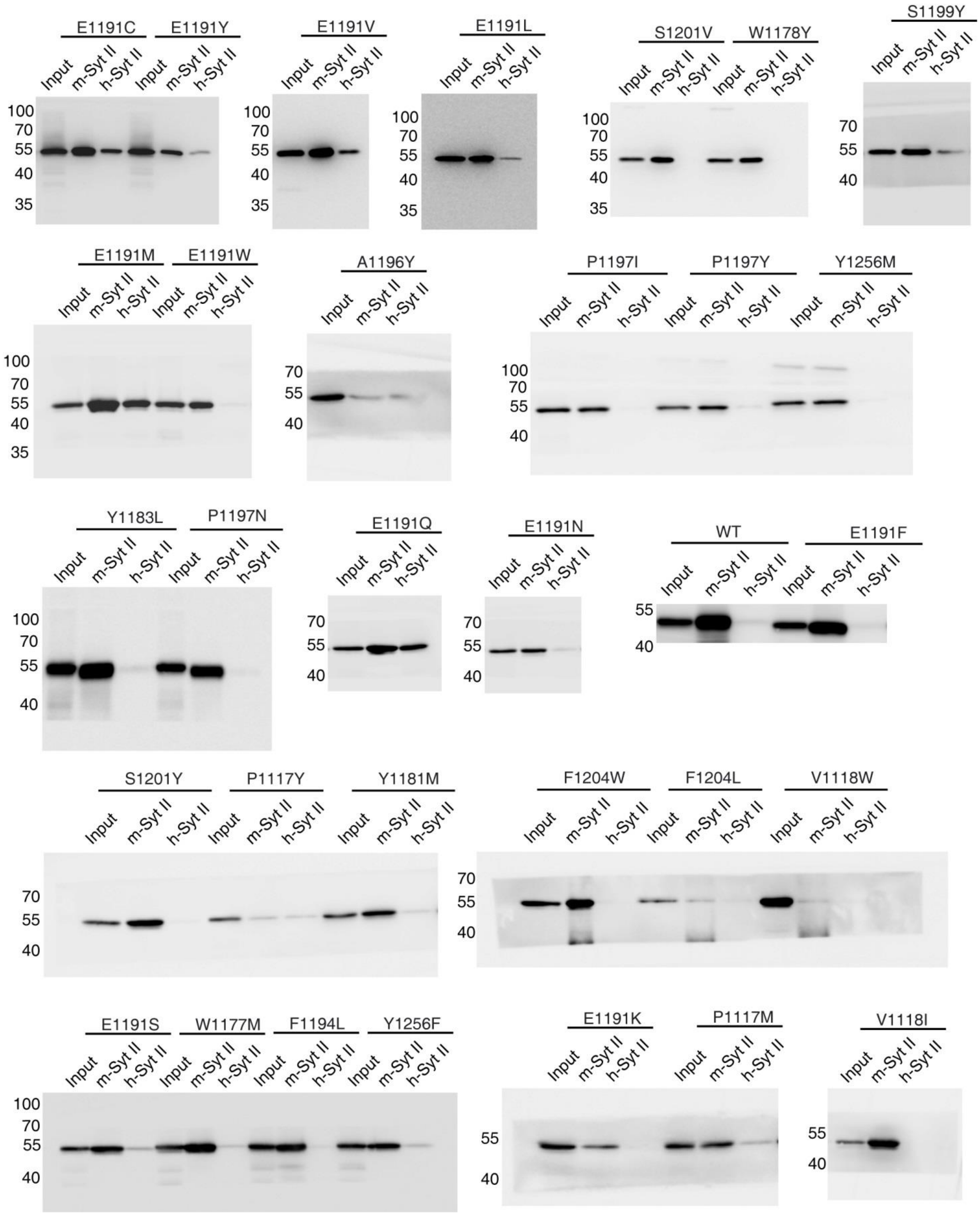

Supplementary Figure 8. Full blot images.

Figure 2a

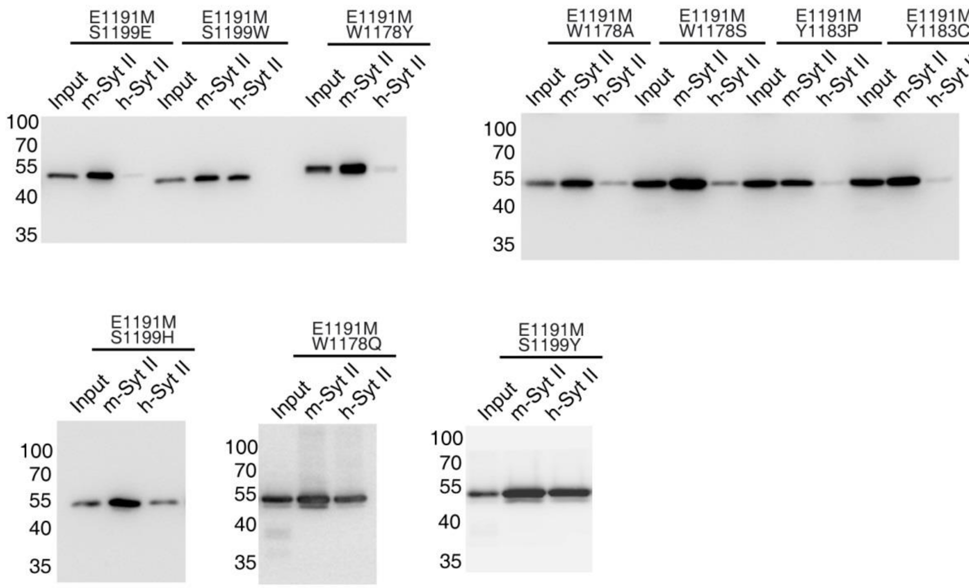

Figure 2c

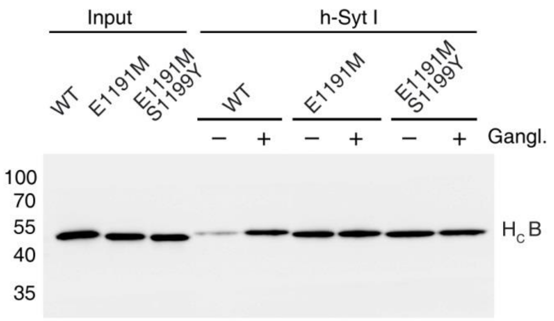

Figure 4a

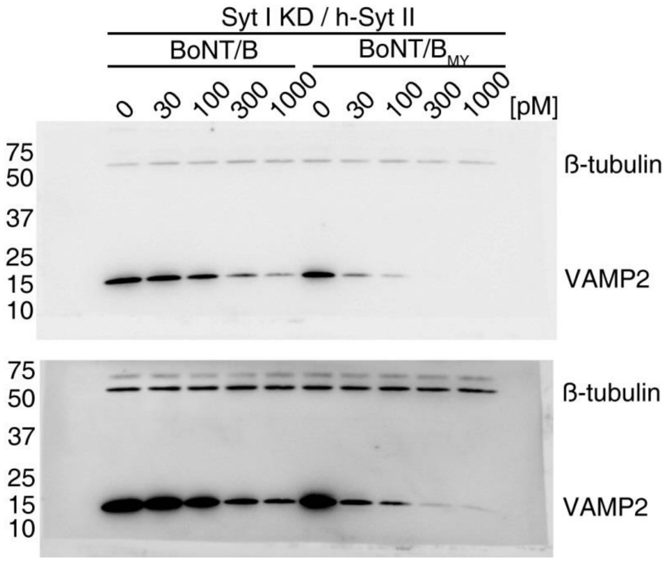

Supplementary Figure 8. Full blot images.

Figure 5b

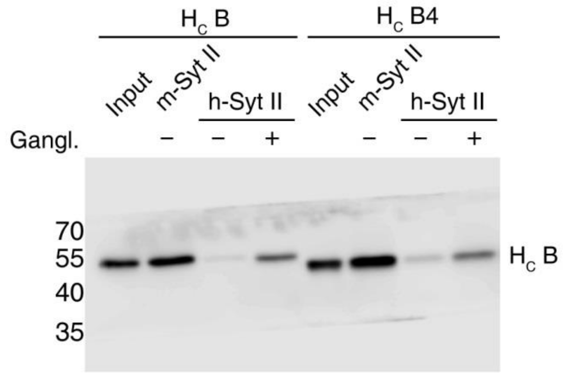

Figure 5c

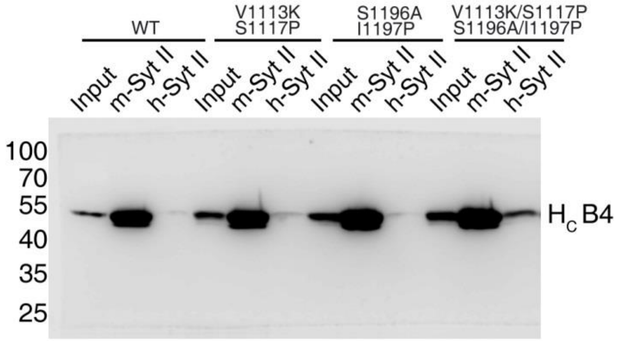

Figure 5d

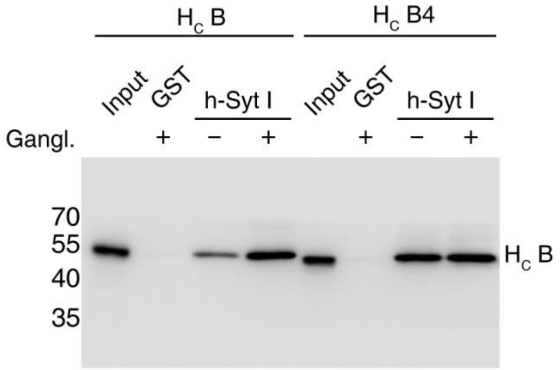

Supplementary Fig. 5

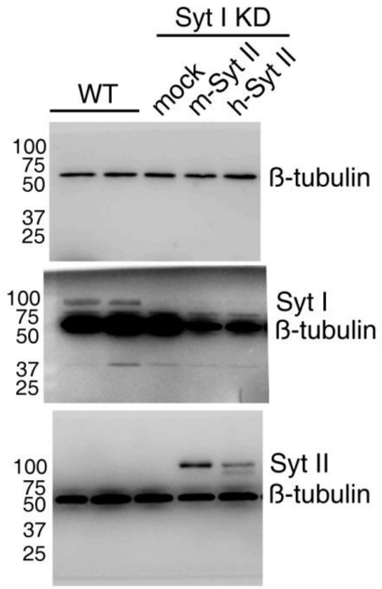

Supplementary Fig. 6

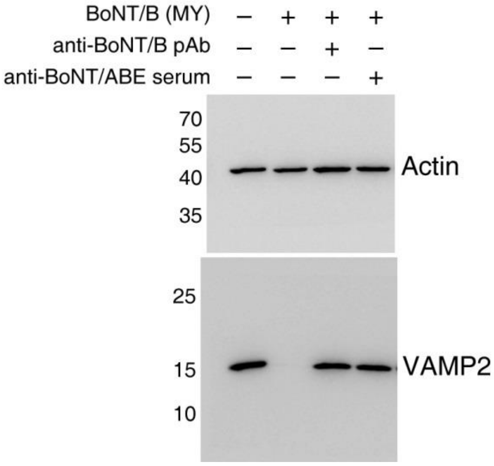

Supplementary Figure 8. Full blot images.

Supplementary Table 1: A list of 19 key residues in BoNT/B that form the Syt-binding pocket, based on co-crystal structures of BoNT/B in complex with Syt II.

Residue in BoNT/B	Close to residue X in Syt II	Residue in BoNT/B	Close to residue X in Syt II
1113	57	1194	47, 50, 51, and 54
1115	53	1196	47 and 50
1116	53 and 57	1197	47 and 50
1117	50, 53 and 54	1199	47
1118	54 and 57	1201	47 and 51
1178	50	1203	51 and 55
1181	47	1204	51, 54 and 55
1183	54 and 55	1245	57
1191	58	1256	57
1192	57		

Supplementary Table 2: Summary of the number of colonies at each mutagenesis site from the BACTH screen.

Residue position in BoNT/B	Number of screened colonies	Number of blue colonies	Percentage of blue colonies
1113	935	0	0
1115	382	0	0
1116	>1000	0	0
1117	>1000	0	0
1118	>1000	0	0
1178	380	29	7.6
1181	440	0	0
1183	980	39	4.0
1191	874	198	22.7
1192	>1000	0	0
1194	620	0	0
1196	>1000	0	0
1197	>1000	0	0
1199	624	32	5.1
1201	634	0	0
1203	612	0	0
1204	764	0	0
1245	394	0	0
1256	488	0	0
